# Divergent coevolutionary trajectories in parent–offspring interactions and discrimination against brood parasites revealed by interspecific cross-fostering

**DOI:** 10.1098/rsos.180189

**Published:** 2018-06-20

**Authors:** Alexandra Capodeanu-Nägler, Elena Ruiz de la Torre, Anne-Katrin Eggert, Scott K. Sakaluk, Sandra Steiger

**Affiliations:** 1 Institute of Evolutionary Ecology and Conservation Genomics, University of Ulm, Ulm, Germany; 2 Faculty of Sciences, University of Málaga, Málaga, Spain; 3 Behavior, Ecology, Evolution and Systematics Section, School of Biological Sciences, Illinois State University, Normal, IL, USA; 4 Institute of Insect Biotechnology, University of Gießen, Gießen, Germany

**Keywords:** parent–offspring interactions, coevolution, burying beetles, *Nicrophorus*, cross-fostering, brood parasitism

## Abstract

In animal families, parents are expected to adapt to their offspring's traits, and offspring, in turn, are expected to adapt to the environment circumscribed by their parents. However, whether such coevolutionary trajectories differ between closely related species is poorly understood. Here, we employ interspecific cross-fostering in three species of burying beetles, *Nicrophorus orbicollis*, *Nicrophorus pustulatus* and *Nicrophorus vespilloides*, to test for divergent co-adaptation among species with different degrees of offspring dependency on parental care, and to test whether they are able to discriminate against interspecific parasites. We found that offspring survival was always higher when offspring were reared by conspecific rather than heterospecific parents. In the case of *N. orbicollis* raising *N. pustulatus*, none of the larvae survived. Overall, these results indicate that parent and offspring traits have diverged between species, and that the differential survival of conspecific and heterospecific larvae is because of improper matching of co-adapted traits, or, in the case of *N. orbicollis* with larval *N. pustulatus*, because of selection on parents to recognize and destroy interspecific brood parasites. We suggest that burying beetles experiencing a high risk of brood parasitism have evolved direct recognition mechanisms that enable them to selectively kill larvae of potential brood parasites.

## 1. Background

Family life often involves intense interactions between parents and offspring, and gives rise to a variety of potential conflicts among family members. Traits that are involved in such interactions, e.g. offspring demand and parental provisioning, are expected to coevolve and may become genetically correlated, ultimately leading to co-adaptation of offspring and parental traits [1–3]. In the process of co-adaptation, combinations of offspring and parental traits that simultaneously maximize the fitness of all family members are favoured, thereby contributing to the resolution of parent–offspring conflict over parental care [2–4]. To test for parent–offspring co-adaptation within families, cross-fostering of whole clutches in a wild population of blue tits revealed a sex-specific co-adaptation in which paternal, but not maternal, responsiveness in provisioning (to changes in brood size) was negatively correlated with begging responsiveness in offspring [5]. Fathers that strongly changed their provisioning rate with brood size reared offspring that exhibited limited variation in the level of begging intensity with food deprivation [5]. This study is one of the few empirical studies showing that co-adaptation between parents and offspring can occur.

Generally, whenever species diverge in their ecology, we expect varying strategies of parental care that influence offspring traits to match current environmental conditions. In turn, owing to coevolutionary interactions between parents and offspring, offspring traits are expected to adapt to the social environment circumscribed by their parents [6], and thus evolve alongside parental carer traits. Species-specific coevolutionary trajectories of family members, however, are difficult to measure, especially as species often differ in many other factors than family life. One possible solution is to combine a common garden-type experiment with an interspecific cross-fostering design using closely related species. Such an experimental design should reveal species differences in parent–offspring co-adaptation if offspring reared by conspecific rather than heterospecific parents experience improved survival or growth. A cross-fostering study of two closely related bird species, the pied and collared flycatcher (*Ficedula hypoleuca* and *Ficedula albicollis*), tested for between-species variation in parent–offspring co-adaptation [7]. Here, young of collared flycatchers begged more intensely than young of pied flycatchers, and experienced a growth advantage when reared by heterospecific foster parents, whereas young of pied flycatchers did not. Although the feeding frequency did not differ between the species of attending parents, the environment created by adult pied flycatchers generally appears to be more beneficial to offspring [7]. Thus, rather than co-adaptation between parents and offspring, variation in offspring traits seems to indicate intrinsic differences in need, which is probably linked to a differentiation of life-history traits [7].

Independent of any potential co-adaptation between parents and young, recognition by parents of their own offspring is an important characteristic of many caring species in which there is a risk that unrelated conspecific or even heterospecific young might be present in their nest or with their brood. Interspecific brood parasitism, as occurs in several bird and hymenopteran species [8], is one context in which caring for unrelated young is maladaptive, such that the ability to discriminate against unrelated young can have substantial fitness benefits for both parents and offspring [9,10]. Recognition of own young can be direct when parents identify phenotypic cues or sets of traits in all of their individual offspring that can be chemical (e.g. [11,12]), acoustic (e.g. [13,14]) or visual in nature (e.g. [15,16]). Parents recognizing individual young could accomplish this via recognition alleles, phenotype matching or associative learning [10,17–19]. In indirect recognition, contextual rather than individual cues are used, resulting in acceptance of all young that are present in a certain location [20] or at a certain time [21]. Empirical studies have documented that parental discrimination against unrelated offspring is present in some species (e.g. [22–24]) and absent in others (e.g. [25–27]), and the most probable cause for this difference is the actual risk of parasitism in natural parent–young interactions.

Here, we tested for parent–offspring co-adaptation and discrimination against heterospecific young by cross-fostering offspring between different burying beetle species of the genus *Nicrophorus*. We manipulated combinations of caring and recipient species, while keeping all other parameters constant across experiments. Burying beetle parents provide extensive biparental care to their offspring before and after hatching [28–31]. Larvae beg for pre-digested carrion, but can also self-feed from the carcass, although parental provisioning has been generally shown to enhance survival and growth of larvae [30,32,33]. Conspecific individuals can easily be cross-fostered, as burying beetles are thought to use primarily temporal cues to recognize their own larvae [21]. A previous cross-fostering study suggested within-family co-adaptation of parent and offspring behaviour in *Nicrophorus vespilloides*: levels of parental provisioning and larval begging were genetically correlated, and matching levels of care and begging resulted in higher offspring fitness [32]. A more recent study using *Nicrophorus orbicollis* and *N. vespilloides*, however, found no evidence for any co-adaptation within species: there was no significant interaction between the effects of caring and recipient species for either larval development time or larval mass [34]. Differences between species could, therefore, be attributed to differences in life-history traits rather than co-adapted parental care [34,35].

In an earlier study, we found that offspring of the three species, *Nicrophorus orbicollis*, *N. pustulatus* and *N. vespilloides*, show striking variation in their reliance on post-hatching care, which consists mainly of food provisioning [33]. The time larvae spent begging and the time parents spent provisioning also differed greatly between the three species, and this aligned closely with the nutritional dependence of offspring: the more-dependent *N. orbicollis* young invested the most time in begging, whereas the less-dependent *N. pustulatus* begged the least [36]. In this study, we employed between-species cross-fostering to measure the degree of co-adaptation of parent and offspring traits among *N. orbicollis*, *N. pustulatus* and *N. vespilloides*. Given that larvae of the three species are so different in their dependence on parental care, we expected to find different degrees of co-adaptation. Specifically, we predicted that fitness differences between offspring reared by conspecific and heterospecific foster parents should be more distinct in *N. orbicollis* than in *N. pustulatus*. Asymmetrical trajectories in parent–offspring interactions might also be expected. Larvae reared by parents showing high levels of provisioning should also benefit from heterospecific parents exhibiting high levels of provisioning, but might suffer from reduced fitness when reared by foster parents showing low levels of provisioning. Larvae from more-dependent species should do better when reared by conspecific parents with matching levels of care, but for larvae of less-dependent species, the species of the carer might be less important. Further, we addressed parental discrimination between conspecific and heterospecific young. Previous studies showed that *N. vespilloides* and *N. orbicollis* parents tolerate each other's larvae [34]; however, it is currently unknown whether the acceptance of congeneric larvae is ubiquitous in *Nicrophorus.* Discrimination against heterospecific young is selectively favoured if interspecific brood parasitism regularly occurs between two species in the field, as suggested for *N. pustulatus* as a brood parasite of *N. orbicollis* [37]. As *N. vespilloides* and *N. pustulatus* originate from allopatric populations on different continents that have no recent evolutionary history with each other, no interspecific recognition would be expected.

Interspecific cross-fostering experiments simultaneously test for both parent–offspring co-adaptation and discrimination against heterospecific larvae, but it can be a major challenge to experimentally disentangle the effects of the two processes because, in both cases, we would predict lower survival when offspring are reared by heterospecific than by conspecific parents. However, burying beetles kill larvae through cannibalism, when they decide not to care for them. This strategy can be observed, for example, when larvae arrive before the expected time of hatching [21] or when parents reduce the brood size to match it to carcass size [38,39]. Thus, we predict that, in the case of discrimination against brood parasites, parents should kill all heterospecific larvae, rather than raising fewer of them. In the case of co-adaptation, however, we predict that some heterospecific larvae should survive, but that larvae of all species should survive and grow best with parents of their own species. These predictions allow us to discriminate between co-adaptation and discrimination in the present study.

## 2. Material and methods

### 2.1. Origin and maintenance of experimental animals

*Nicrophorus vespilloides* used in the experiment were descendants of beetles collected from carrion-baited pitfall traps in a forest near Ulm, Germany (48°25′03^^″^^ N, 9°57′45^″^ E). Colonies of *N. pustulatus* and *N. orbicollis* were established at Ulm University from outbred colonies maintained in the Institute of Zoology at the University of Freiburg, Germany. We maintained outbred colonies of both species by introducing beetles captured in baited pitfall traps established in a forested area near Lexington, Illinois, USA (40°39′57^″^ N, 88°53′49^″^ W). Both American species are sympatric, but occur allopatric to the population of *N. vespilloides* we used. All beetles were kept in temperature-controlled chambers at 20°C under a 16 L : 8 D cycle. Before the experiments, groups of up to five same-sex siblings of each species were kept in small transparent plastic containers (10 × 10 cm and 6 cm high) filled with moist peat. Beetles were fed freshly decapitated mealworms ad libitum twice a week. At the time of experiments, beetles were virgin and between 25 and 40 days of age.

### 2.2. Experimental design

#### 2.2.1. General procedures and cross-fostering

In each species, we randomly paired non-sibling beetles and induced reproduction by providing them with a 20 g (±3 g) thawed mouse carcass (Frostfutter.de – B.A.F Group GmbH, Germany). In the case of the nocturnal species, *N. pustulatus* and *N. orbicollis*, mice were provided during the dark portion of the photoperiod. Because the developmental time from egg laying to the hatching of larvae is shorter in *N. vespilloides*, pairs of *N. orbicollis* and *N. pustulatus* were set up one day earlier to ensure simultaneous larval hatching. After the egg-laying period, but before hatching (see [33]), parents and the carcass were transferred to new plastic containers filled with soil. The old containers containing the eggs were checked every 8 h for the presence of newly hatched larvae. Upon hatching, larvae were pooled to control for within-family variation and individual differences, and kept in a Petri dish with moist filter paper at 4°C if not used in experiments immediately. Clutch size varies greatly within and among species, and could, therefore, have an influence on brood size and larval weight. Thus, we pooled 15 larvae of the same species to create broods of mixed parentage in each species and treatment, which is standard procedure in burying beetle studies [33,40–43]. We performed crosses between *N. orbicollis* and *N. pustulatus*, and between *N. pustulatus* and *N. vespilloides*. As cross-fostering experiments between *N. orbicolli*s and *N. vespilloides* were conducted previously [34], we opted to omit this combination in our study. We established four different treatment groups (parents were provided with heterospecific offspring) plus one control group for each treatment (parents were provided with conspecific offspring) to compare parenting behaviour of individuals towards conspecific or heterospecific offspring (table 1). Because we used *N. pustulatus* twice in combination with the two other species, we established a control group for each combination.

**Table 1.** Cross-fostering combinations of parent and offspring species and sample sizes. (Parents were either provided with 15 heterospecific or conspecific larvae (control). NO, *N. orbicollis*; NP, *N. pustulatus*; and NV, *N. vespilloides*.)

	parents							
	*N. orbicollis*			*N. pustulatus*			*N. vespilloides*	
offspring	NO	NP	NP	NO	NP	NV	NV	NP
sample size	17	15	15	15	16	18	21	21

Larvae were added directly on top of the carcass, which we had sliced open to allow larvae in each treatment to access the carrion more easily. Females exhibit temporally based kin discrimination, in which they kill any larvae arriving on the carcass before their own larvae would have hatched [21]. Thus, we only provided pairs with larvae after their own larvae had started hatching. During the first 2 days after adding the larvae, we monitored broods every 4 h to check whether larvae were alive and cared for by the foster parents. If we could not observe any larvae alive, we gently opened the carcass and inspected the cavity for larvae. When fewer larvae than usual arrive at the feeding cavity, the parents occasionally reclose the carcass, causing larvae to suffocate inside, and resume mating and egg laying [29,44]. In these instances, larval stimuli (perhaps offspring begging) appear to be insufficient to trigger and maintain parental care behaviour [45]. Alternatively, if we did not find any larvae in the surrounding soil or inside the carcass, we assumed that parents had actively killed the larvae, which happens regularly during brood size regulation [38,39,46] and in time-based kin recognition [21]. We monitored broods twice a day for the dispersal of larvae. At the time of dispersal, surviving larvae were weighed and counted.

#### 2.2.2. Survival of larval *Nicrophorus pustulatus* when reared in pure or mixed-species broods by parental *Nicrophorus orbicollis*

Upon discovering that none of the larval *N. pustulatus* survived when reared by *N. orbicollis*, we conducted further experiments to confirm that this is the outcome of active discrimination against heterospecific larvae. Alternatively, the reduced begging behaviour of *N. pustulatus* larvae [36] might be insufficient to trigger parental care behaviour in *N. orbicollis,* which also might result in parents killing the larvae. To distinguish between these two possibilities, we established broods in which *N. orbicollis* parents experienced an increased begging stimulus. This was done by providing *N. orbicollis* with an increased number of *N. pustulatus* larvae or by providing them with mixed broods consisting of conspecific (highly begging larvae) and heterospecific larvae. Thus, we established three treatment groups in which we provided *N. orbicollis* parents with: (i) 30 *N. pustulatus* larvae to test whether the begging frequency experienced by parents played a role (*n* = 11); (ii) a mixed brood consisting of eight *N. orbicollis* and eight *N. pustulatus* larvae (*n* = 15) to assess whether mixed broods made acceptance of heterospecific larvae more likely; and (iii) a mixed brood consisting of three *N. orbicollis* and 13 *N. pustulatus* larvae (*n* = 10) to assess whether the presence of only a few *N. orbicollis* larvae was sufficient to ensure the survival of *N. pustulatus* in the same brood. Experimental procedures for these treatments were the same as in the first experiment. When larvae had left a carcass, they were counted, weighed and transferred into new plastic containers with moist peat. For treatments with mixed broods, we determined species identity after adult emergence. Survival from larval dispersal to adult emergence was generally high, and only 11 larvae did not complete development to adulthood.

### 2.3. Statistical analyses

All data (see the electronic supplementary material) were analysed and plotted using R v. 3.1.2 [47]. Our experimental procedure yielded two datasets, each reflecting a 2 × 2 factorial design. The first dataset contains crosses between *N. orbicollis* and *N. pustulatus*, whereas the second dataset contains crosses between *N. pustulatus* and *N. vespilloides*. In each of these datasets, we used generalized linear models (GLMs) followed by *post hoc* Tukey comparisons, with parent and offspring species as fixed factors, and the number of larvae surviving and mean larval mass per brood as dependent variables. As the clutches were standardized to 15 larvae, we used the absolute number of larvae that survived. We then applied GLMs with a quasi-Poisson distribution. To compare mean larval masses per brood between offspring of the different species, we used GLMs with a Gaussian distribution. For the second part of our experiments, we used a Wilcoxon signed-rank test to compare the proportion of larvae that survived in the mixed broods.

## 3. Results

### 3.1. Cross-fostering between *Nicrophorus orbicollis* and *Nicrophorus pustulatus*

**Figure 1.**
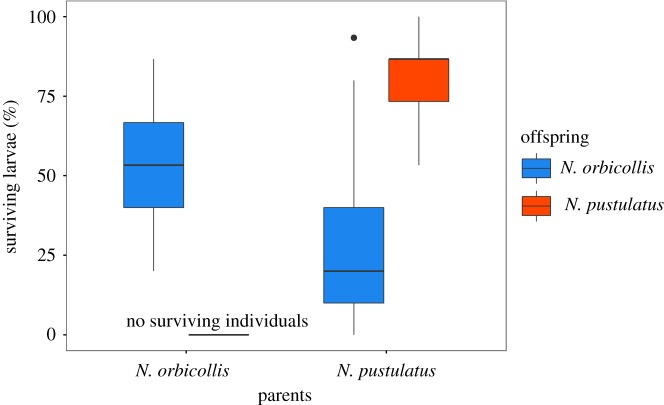
Per cent of larvae surviving to dispersal. Blue bars represent *N. orbicollis* offspring; red bars represent *N. pustulatus* offspring. Boxplots show median, interquartile range, minimum/maximum range. Points are values that fall outside the interquartile range (greater than 1.5× interquartile range).

In the cross-foster experiments involving *N. orbicollis* and *N. pustulatus*, both the caring species (GLM with quasi-Poisson errors: *F*_1,58_ = 28.44, *p *< 0.001, figure 1) and the interaction between caring and recipient species (*F*_1,58_ = 162.88, *p *< 0.001) significantly affected larval survival, but the recipient species alone did not (*F*_1,58_ = 0.08, *p *= 0.78). Significantly fewer *N. orbicollis* offspring survived when reared by *N. pustulatus*, than when reared by conspecific parents (Tukey's *post hoc* test: *p *= 0.002). Under the care of *N. pustulatus*, significantly more conspecific than heterospecific larvae survived (Tukey's *post hoc* test: *p *< 0.001). With *N. orbicollis* parents, survival differences were even more dramatic: not a single *N. pustulatus* larva survived in the 15 broods tested, while more than half of conspecific larvae survived (figure 1). In the control groups with matching species of parent and offspring, larval survival was higher in *N. pustulatus* than in *N. orbicollis* (Tukey's *post hoc* test: *p *= 0.006).

**Figure 2.**
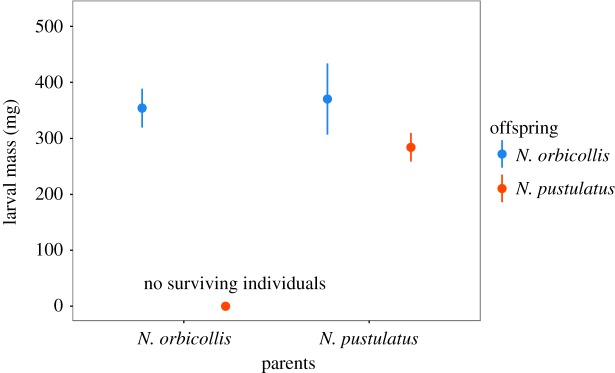
Mass (milligram) of larvae surviving to dispersal. Blue means represent *N. orbicollis* offspring; red means represent *N. pustulatus* offspring. Plots show the mean ± 95% confidence intervals.

Because none of the *N. pustulatus* larvae survived when *N. orbicollis* was the caring species, we tested differences in larval weight using a one-way GLM including the remaining three treatment levels. Generally, we found that larval masses differed significantly between the treatments (GLM with Gaussian errors: *F*_2,42_ = 5.45, *p *= 0.008; figure 2). *Nicrophorus orbicollis* larvae were significantly heavier than *N. pustulatus* larvae, both when *N. pustulatus* was the caring species (Tukey's *post hoc* test: *p *= 0.01) and in the control groups (Tukey's *post hoc* test: *p *= 0.03).

### 3.2. Cross-fostering between *Nicrophorus pustulatus* and *Nicrophorus vespilloides*

**Figure 3.**
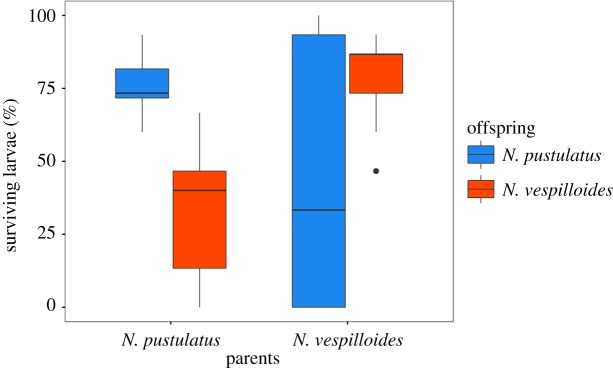
Per cent of larvae surviving to dispersal. Blue bars represent *N. pustulatus* offspring; red bars represent *N. vespilloides* offspring. Boxplots show median, interquartile range, minimum/maximum range. Points are values that fall outside the interquartile range (greater than 1.5× interquartile range).

In this combination, neither the caring species (GLM with quasi-Poisson errors: *F*_1,72_ = 2.07, *p *= 0.15; figure 3) nor the recipient species (*F*_1,72_ = 0.0004, *p *= 0.98) had a significant effect on larval survival, but the interaction between the caring and recipient species did (*F*_1,72_ = 32.19, *p *< 0.001). When *N. pustulatus* was the caring species, survival of *N. vespilloides* larvae was significantly lower than that of *N. pustulatus* larvae (Tukey's *post hoc* test: *p *< 0.001), but when *N. vespilloides* was the caring species, more *N. vespilloides* than *N. pustulatus* larvae survived (Tukey's *post hoc* test: *p *= 0.002). For both species of larvae, survival was higher when reared by conspecific than when reared by heterospecific parents (Tukey's *post hoc* tests, *N. pustulatus* larvae: *p *= 0.02, *N. vespilloides* larvae: *p* < 0.001).

**Figure 4.**
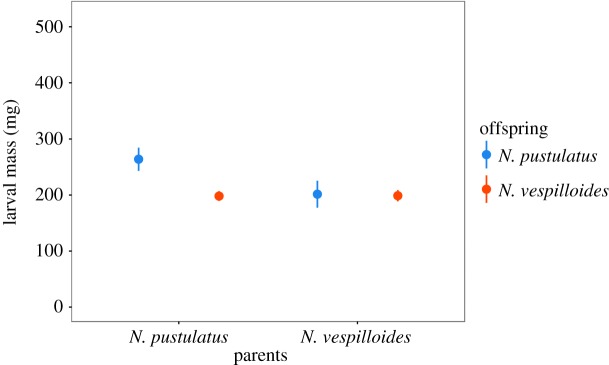
Mass (milligram) of larvae surviving to dispersal. Blue means represent *N. pustulatus* offspring; red means represent *N. vespilloides* offspring. Plots show the mean ± 95% confidence intervals.

We found significant effects of caring species (GLM with Gaussian errors: *F*_1,61_ = 13.50, *p *< 0.001; figure 4) and recipient species (*F*_1,61_ = 19.49, *p *< 0.001) as well as a significant interaction between the two (*F*_1,61_ = 17.05, *p *< 0.001) on larval mass at dispersal. In the control treatments with conspecific parents, *N. pustulatus* larvae were heavier than *N. vespilloides* larvae (Tukey's *post hoc* test: *p *< 0.001), and the same was true for the larvae that were reared by *N. pustulatus* (Tukey's *post hoc* test: *p *< 0.001). With *N. vespilloides* parents, however, surviving larvae of both species reached approximately the same size. Consequently, the final mass of *N. pustulatus* larvae was lower when they were reared by *N. vespilloides* (Tukey's *post hoc* test: *p *< 0.001), but the final mass of *N. vespilloides* larvae was not affected by the caring species.

### 3.3. Survival of larval *Nicrophorus pustulatus* when reared in pure or mixed-species broods by parental *Nicrophorus orbicollis*

**Figure 5.**
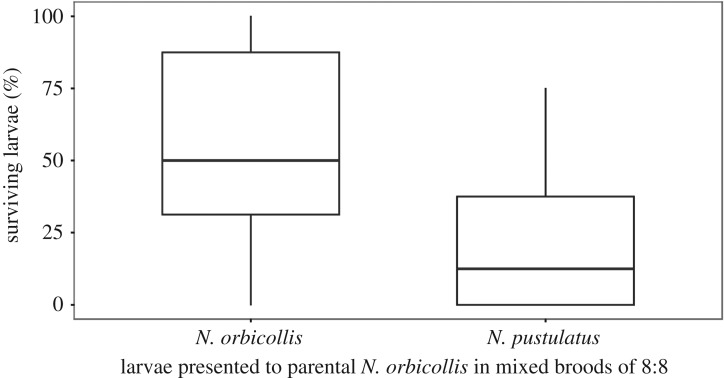
Per cent of larvae surviving to dispersal in mixed broods (eight *N. orbicollis*; eight *N. pustulatus*) with *N. orbicollis* parents. Boxplots show median, interquartile range, minimum/maximum range.

No larvae survived in any of the broods in which we provided parental *N. orbicollis* with 30 *N. pustulatus* larvae. For these broods, we observed that *N. orbicollis* parents reclosed the carcass no later than 4 h after larvae had been added. When we inspected those breeding boxes immediately thereafter to search for the missing larvae, we did not find any remains or dead larvae, neither in the surrounding soil, nor inside the carcass that we had opened. When parental *N. orbicollis* were provided with mixed broods comprising equal numbers of *N. orbicollis* and *N. pustulatus* larvae, some of the *N. pustulatus* survived (median: 1, 1. quartile: 0, 3. quartile: 3), but significantly fewer than of the *N. orbicollis* larvae (median: 4, 1. quartile: 2.5, 3. quartile: 7) (Wilcoxon signed-rank test: *V* = 99.5, *p *= 0.026; figure 5). When broods consisted of 13 *N. pustulatus* and three conspecific larvae instead, none of the larvae survived in six of 10 broods. In the four surviving broods, on average, 2.5 (median: 2.5, 1. quartile: 2, 3. quartile: 3) *N. orbicollis* larvae survived, but only a single *N. pustulatus* larva in one brood survived.

## 4. Discussion

Our previous work has shown that parenting strategies and the degree of offspring reliance on parental care has diverged significantly in the three burying beetle species *N. orbicollis*, *N. pustulatus* and *N. vespilloides*. In this study, we used the same species and explored the consequences of creating a mismatch of parent and offspring traits by providing parents with heterospecific larvae. We found that larval survival was always lower when they were reared by heterospecific than by conspecific parents, even though initial brood size was kept constant. Our data indicate that a portion of these results can indeed be explained by parent–offspring co-adaptation. Parental and offspring traits have diverged between species, but coevolved within species, suggesting that offspring traits have specifically adapted to the environment provided by their parents, and parental traits to the environment of their offspring. However, larvae from species with low provisioning rates did not appear to benefit when reared by parents with high levels of provisioning, suggesting that parental care and its effects are not easily quantified, and that the coevolved match between parents and offspring involves components beyond simple feeding rates. Moreover, we also found evidence that at least *N. orbicollis* has the ability to discriminate against heterospecific larvae. In the combination of *N. orbicollis* parents with *N. pustulatus* larvae, none of the larvae survived, which suggests that the parents actively killed all larvae, probably owing to the high risk of brood parasitism.

The significant interaction terms between caring and recipient species for both larval survival and larval mass suggest that within-species coevolution between parental and offspring traits has occurred that increases fitness benefits of parental care. This is supported by our results showing that, in all three species, larvae suffered reduced survival when reared by heterospecific parents compared with offspring reared by conspecific parents. By contrast, in a cross-fostering experiment involving *N. orbicollis* and *N. vespilloides*, Benowitz *et al*. [34] found no evidence of species-specific co-adaptation between offspring and maternal traits (no significant interactions between caretaker and recipient species for development time or larval mass) and attributed differences in caring to life-history differences between species. The discrepancy between the results of their study and those of this study could be owing to the fact that Benowitz *et al*. [34] did not standardize initial brood size, nor did they examine offspring survival rates. Variation in brood size can influence larval growth and development (e.g. [38,39,46,48]), and thus obscure any apparent co-adaptation. Alternatively, *N. orbicollis* and *N vespilloides* might resemble each other more than the combinations of species we used.

Based on our previous study [36], we expected negative effects of rearing by heterospecific parents to be more pronounced in young that are more dependent on parental care and beg more (*N. orbicollis* and *N. vespilloides*), than in young that are less dependent (*N. pustulatus*). However, this hypothesis was not confirmed by the results of the current study. Some of the highly dependent *N. orbicollis* larvae were even reared by parents of the most independent species, *N. pustulatus*, although their survival was lower than when reared by conspecific parents. The amount and value of care provided by *N. pustulatus* was low in intraspecific interactions [36], but it is conceivable that larval *N. orbicollis* are able to manipulate the feeding behaviour of parental *N. pustulatus* by begging more intensely. Perhaps more surprisingly, it was the independent larvae of *N. pustulatus* that experienced the greatest fitness loss with heterospecific parents, as no larvae were reared by *N. orbicollis* and survival was reduced when reared by *N. vespilloides*. In the case of *N. orbicollis* rearing *N. pustulatus*, however, this result is probably the result of discrimination against brood parasites and not co-adaptation, as *N. orbicollis* parents seem to have actively killed all heterospecific larvae. In the case of *N. pustulatus* rearing *N. vespilloides* larvae, some larvae survived, which argues against discrimination and active larval killing. Interestingly, the *N. pustulatus* larvae grew less well when reared by *N. vespilloides* than when reared by conspecifics, although feeding rates of parental *N. vespilloides* are higher than feeding rates of parental *N. pustulatus* [36]. A potential explanation for this finding might lie in the species-specific microbiome that is transferred from parents to the carcass surface and the larvae via oral and anal secretions [49,50]. Recent studies provide evidence of potential metabolic cooperation between the burying beetle host and its microbiota for digestion, detoxification and defence [51]. Hence, the transfer of an unsuitable microbiome, adapted to the offspring traits of *N. vespilloides*, rather than a deficiency in provisioning, might explain the fitness disadvantage of *N. pustulatus* reared by *N. vespilloides*. Alternatively, the oral secretions of parents that are transferred to the offspring during feeding bouts may comprise a mixture of compounds that have adapted to match the needs of conspecific, but not necessarily heterospecific offspring. In a recent study, LeBoeuf *et al*. [52] revealed that insect oral fluids can contain a huge variety of chemical compounds and effector molecules, such as species-specific growth-regulatory proteins and hormones that are essential for the survival and development of offspring. Thus, oral secretions of parents that are beneficial to *N. vespilloides* might negatively affect fitness of larval *N. pustulatus*. However, we should note that these two species appear to use different food sources in nature, as *N. pustulatus* has been frequently found to exploit snake eggs [53,54]. Thus, an interspecific mismatch with respect to a species-specific microbiome or oral secretions may not apply to *Nicrophorus* species that use small vertebrate carcasses as a food source for their larvae.

The lower survival and growth rate of larvae reared by heterospecific parents could also be the result of a mismatch of produced signals and receiver response. The communication and interaction between parents and larvae should match to trigger and maintain parental care behaviours. Interactions between parents and offspring are stronger in *N. orbicollis* and *N. vespilloides* than in *N. pustulatus*, as larvae of both species beg more and parents provision more than caring *N. pustulatus* [36]. Thus, close relationships between parents and offspring might allow for a species-specific behaviour of parents that have adapted to respond to the signals of conspecific offspring. In burying beetles, larvae are known to beg when parents are present [43,55] and parents respond to begging by providing food to the larvae [55,56]. Thus, if it is the amount of begging by larvae that provides information about the presence of young and triggers the onset of parental care [45], the begging stimulus of *N. pustulatus* might be insufficient to achieve the same amount of care as when cared for by conspecific parents that have adapted to respond to low amounts of begging. For example, Bell's vireo parents (*Vireo bellii*) feed single foreign nestlings at a rate that is significantly less than their feeding rate for a typical brood of their own offspring [57]. Here, parents do not respond to begging of foreign offspring in the same way as to their own offspring, resulting in lighter and smaller foreign young. Rivers *et al*. [57] concluded that one single foreign nestling provides an inadequate stimulus for vireo parents, presumably because of a mismatch between begging displays of foreign and conspecific offspring. For the combination of *N. orbicollis* and *N. pustulatus* in our study, however, even 30 larval *N. pustulatus* were not sufficient to trigger any parental care in *N. orbicollis*. Thus, we suggest that rather than a mismatch between the caring and the recipient species, parental *N. orbicollis* directly discriminated against larval *N. pustulatus* by actively cannibalizing all larvae, resulting in total brood losses.

However, why did *N. orbicollis* only discriminate against *N. pustulatus* larvae, whereas in all other combinations, heterospecific larvae were accepted and raised? Generally, it is likely that the ability of certain species to discriminate against larvae of certain others is because of stronger selection for this ability. Discrimination against unrelated young (heterospecific or conspecific) by caring parents is usually expected to avoid wasting parental time, energy and resources for misdirected care. If there is a risk of brood parasitism, selection for such discrimination will occur because parasitism usually reduces a host's reproductive output [8,58]. In birds, for example, fairy wren hosts (*Malurus cyaneus*) that are exposed to high levels of parasitism and that had experience with cuckoos in the past show intense mobbing behaviour towards two species of parasitic bronze-cuckoos, whereas rarely parasitized and inexperienced hosts show little reaction [59]. Selection for interspecific recognition might be especially strong on *Nicrophorus orbicollis*, which co-occurs with *N. pustulatus* in large parts of its range and in the same woodland habitats, and is reproductively active at the same time [31]. In the laboratory, female *N. pustulatus* can successfully parasitize broods of *N. orbicollis*, and produce very large clutches that could easily swamp the broods of other species [37]. This could explain the adaptive benefit of *N. orbicollis*' near-perfect discrimination against larval *N. pustulatus*, which may be a result of previous exposure to parasitism pressure. Another species, *Nicrophorus tomentosus*, has also been found in the field on a carcass with *N. orbicollis* in residence [60], suggesting that the threat of interspecific parasitism may be especially high for *N. orbicollis*. Although multiple species of burying beetles occur in most habitats where they have been studied [28,29,31,61], and most of them use similar resources, discrimination against interspecific brood parasites should be especially beneficial for those species or populations that experience a high risk of such parasitism [62]. In our study, we would expect the greatest selection for discrimination in *N. orbicollis* parents with *N. pustulatus* larvae, whereas we have no clear expectation for *N. vespilloides* and *N. pustulatus* because the two species do not usually co-occur and the two source populations for our beetles came from different continents.

While the ultimate function of discrimination against parasites is self-evident, the proximate mechanism used by parents is still obscure. When we created mixed broods with equal numbers of *N. orbicollis* and *N. pustulatus* larvae, fewer *N. pustulatus* than *N. orbicollis* larvae survived. This suggests that the killing of larvae is selective and not a generalized response to overall begging levels in entire broods. As it is highly unlikely that parents can monitor individual larvae for begging rates, they must use direct cues other than larval begging behaviour in the recognition of brood parasites. These cues could be behavioural, morphological, or visual, but it appears most probable that they are chemical in nature because burying beetles use chemical cues in a variety of social contexts [63–69]. The acceptance of *N. pustulatus* was highest in mixed broods with equal numbers of *N. pustulatus* and *N. orbicollis*. This suggests that the more *N. orbicollis* parents are exposed to conspecific larvae, the more likely it is that they accept *N. pustulatus* larvae. In the presence of large numbers of species-appropriate cues, parents may be less discriminating to avoid unnecessary killing of own larvae. Alternatively, the mixing of *N. pustulatus* and *N. orbicollis* larvae may have led to a transfer of cues between species, making it impossible for parents to discriminate against individual larvae.

In summary, we suggest that our findings in the crosses between *N. vespilloides* and *N. pustulatus*, and between *N. pustulatus* parents and *N. orbicollis* offspring are the outcome of co-adaptation. Here, offspring experienced greater fitness loss when reared by heterospecific than by conspecific parents, indicating distinct coevolutionary trajectories. Hence, fitness in the recipient species was dependent on the caring species. We suggest that, at least for these combinations of species used, parenting or communication mechanisms have diverged, but that, within each species, parent–offspring interactions reflect adaptive integration of complementary parental and offspring traits. However, our data also indicate that counter-adaptations to brood parasitism contribute to the differential survival of larvae. We found clear differences in the acceptance of heterospecific offspring among the three *Nicrophorus* species. We suggest that beetles can directly discriminate against heterospecific offspring according to the potential risk of parasitism by another species. This result was most evident in the combination of *N. orbicollis* parents with *N. pustulatus* offspring, in which all heterospecific larvae were killed, and is thus explained best by discrimination. Although we attribute the majority of our results to co-adaptation processes, they do not preclude the possibility that our findings may be the outcome of improper or incomplete discrimination against heterospecific species. Our study highlights the potential benefit of examining recognition mechanisms in greater detail, and of directly observing how parents react towards heterospecific larvae. As highlighted by the review of Royle *et al*. [70], parental care is a coevolving game for the whole family, and we stress that more multi-species and comparative studies are needed to better understand the evolution of different coevolutionary trajectories between the family members and the causation of divergence in parenting strategies and offspring traits.

## Supplementary Material

Supplementary Material - Dataset

## Data accessibility

The dataset supporting this article has been uploaded as part of the electronic supplementary material.
